# Bacterial biofilm formation inside colonic crypts may accelerate colorectal carcinogenesis

**DOI:** 10.1186/s40169-018-0209-2

**Published:** 2018-09-17

**Authors:** Hans Raskov, Kasper Nørskov Kragh, Thomas Bjarnsholt, Mahdi Alamili, Ismail Gögenur

**Affiliations:** 1 Center of Surgical Science, Zealand University Hospital, Lykkebækvej 1, 4600 Koege, Denmark; 2 Costerton Biofilm Center, Department of Immunology & Microbiology, Faculty of Health and Medical Sciences, University of Copenhagen, Blegdamsvej 3B, 2200 Copenhagen, Denmark; 3 Department of Clinical Microbiology, Rigshospitalet, Juliane Maries vej 22, 2100 Copenhagen, Denmark; 4 Gastrounit, Surgical Division, Hvidovre University Hospital, Kettegård Allé 30, 2650 Hvidovre, Denmark; 5 Lundevangsvej 23, 2900 Hellerup, Denmark

**Keywords:** Bacterial biofilm, Colonic crypts, Polyp, Colorectal cancer, Carcinogenesis

## Abstract

**Background:**

Research in the field of relation between microbes and colorectal carcinogenesis has gained increasing interest in past years. Recently, link between microbial biofilm and carcinogenesis in colon was demonstrated by several authors indicating that biofilm not only is a key player in carcinogenesis, but also may contribute to the understanding of side-specific colon cancer—right sided colon cancer versus left sided. In this article, we briefly highlight the major findings of the research of biofilm and carcinogenesis and demonstrate our findings of colonic cancer tissue and colonic polyp examined for biofilm.

**Case presentation:**

Colonic cancer tissue from a patient with a right-sided colon cancer, and an adenoma tubular polyp were examined for biofilm formation by flourescens in situ hybridization. In cancer tissue we found biofilm formation on the surface epithelium but surprisingly also deep into the crypts. No biofilms were found in tubular polyp tissue.

**Conclusions:**

To our knowledge, this is the first-time biofilm formation deep into colonic crypts are demonstrated in a patient with right-sided colon cancer. This may indicate that bacterial biofilm may have a key role in carcinogenesis.

## Background

Interactions between the human host and the microbiota is in focus as accumulating data point towards important links in colorectal carcinogenesis. Experimental data show how potential colorectal cancer (CRC) driver bacteria such as enterotoxigenic *Bacteroides fragilis* (ETBF), *Fusobacterium nucleatum*, colibactin-producing *Escherichia coli* (CPEC) and *Porphyromonas,* damage mucosal barrier functions by degrading mucins, damaging intercellular junctions and cellular DNA. Direct bacterial actions and immunological responses may induce dysplasia and carcinogenesis [1, 2].

A recent study from Dejea et al. report colonization of aggregated bacteria (biofilms) consisting of ETBF and CPEC on the colonic epithelial surface in patients with familial adenomatous polyposis coli (FAP) [3]. The adenomatous polyposis coli (APC) frameshift mutation inherited in FAP (and also found in most sporadic CRC), mimics a stop codon resulting in truncation of the APC protein. The truncated protein prevents free cytoplasmic β-catenin from binding to the intracellular domain of the trans-membranous cadherin receptor thereby blocking the assembly of the E-cadherin/catenin complex. Free β-catenin translocate from the cytoplasm to the cell nucleus stimulating Wnt-signaling and accelerating cell proliferation. Also, dysfunctional cadherin junctions increase the risk of paracellular passageways for microbes.

Dejea’s data suggest that the colonization with ETBF and CPEC, found in more than 50% of the FAP patients examined (in contrast to less than 25% of controls), degrades mucins, destroys cadherins, increases stem cell proliferation through the Wnt-pathway and thereby increasing risk of mutations and CRC in early life.

The bi-layered mucus lining covering the colonic epithelium is the first line of defense separating microbes from epithelial cells and more importantly keeping them away from proliferating stem cells and progenitors (transit amplifying cells) in the lower crypt compartment. The mucin filling the colonic crypts contains antimicrobial peptides such as defensins, cathelicidins and IgA produced by crypt cells and submucosal immune cells. Furthermore, crypt entrances are guarded by sentinel goblet cells ready to flush microbes away by emptying their entire loads of mucins and cytoplasmic contents by exocytosis when the recognition receptors are triggered by pathogen-associated molecular patterns.

The main constituent of the gel-forming mucus layer is the MUC2 protein secreted by goblet cells. Glycosylated MUC2 mucin molecules form a double-layered mucus membrane of multiple, large insoluble net-like structures. The innermost mucus layer covering the epithelial cell surfaces and filling the crypts is densely packed and impermeable to bacteria. This layer is anchored to the epithelium by goblet cells and by trans-membranous mucins on the apical surface of enterocytes [4].

As mucin production pushes mucus luminally, it is exposed to proteolytic activities by the host and by commensal bacterial proteases and glycosidases. The mucus breaks up and expands 2–3 times in volume providing an ideal niche for bacterial colonization and at the same time serving as a giant glycan food source for microbes [5]. This outer layer harbors the commensal microbiota and as it detaches from the dense, inner layer, it moves along with the fecal stream.

Thus, during eubiosis the inner mucus layer and the colonic crypts are free from microorganisms. Bacterial biofilm formation in the inner mucus layer may down-regulate MUC2 mucins and production of defense peptides. This may result in direct contact between bacteria and epithelial cells, which is considered pathological [6].

Dejea et al. report findings of bacterial biofilm formation on the surface of colonic epithelium in nearly all (89%) examined cases of right-sided colon cancer, whereas only a few percent (12%) of the left-sided cancers were biofilm positive. There was 100% concordance between biofilm-positivity on tumor tissue and the colon tissue at the resection margin. A subsequent metabolomics analysis revealed that biofilm formation in the colon significantly contributes to a pro-oncogenic state [6].

Except from the stem cells and progenitors in the lower crypt compartment, differentiated crypt cells (enterocytes, goblet cells and entero-endocrine cells) migrate towards the surface where they are exfoliated. Stem cells and progenitors constitute a dynamic cell population exhibiting plasticity by transitioning between stem and non-stem cell function, heavily influenced by the micro-environment and tissue damage. Stem cells and progenitors can accumulate genetic and epigenetic alterations resulting in increased self-renewal, survival pressure and dysplasia.

**Fig. 1:**
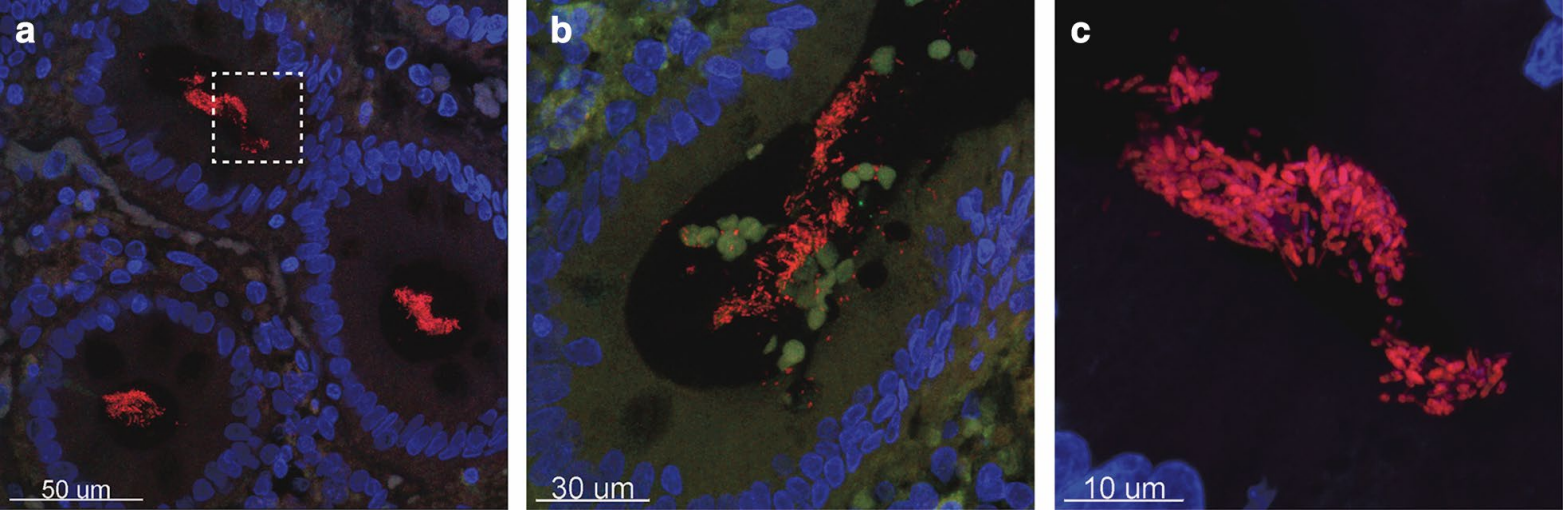
“Top down” (**a**) and “side” (**b**) picture of the colonic crypt in patients with right sided colon cancer. **c** Shows a close up of a bacterial biofilm located in the dashed box in image (**a**). The bacterial biofilm is stained with a universal bacterial PNA FISH probe (red) and nuclei of human cells are counter stained with DAPI (Blue). ×630 magnification

**Fig. 2:**
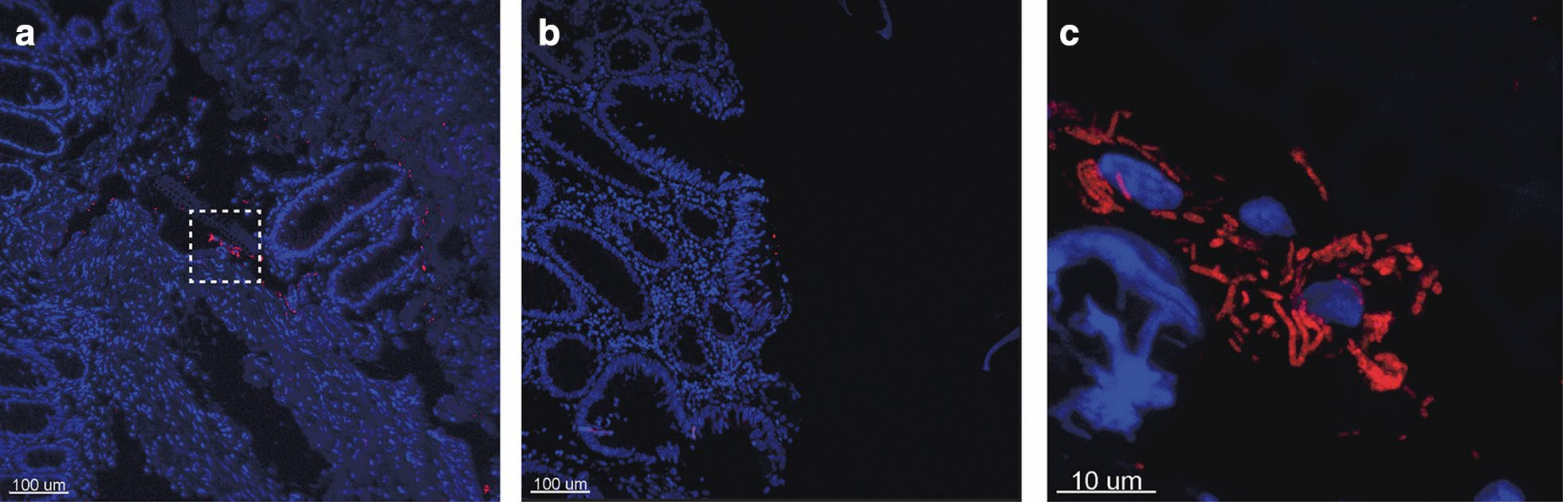
Picture of colonic crypt in patient with colonic tubular adenoma. No sign of biofilm within the crypts. **a** Top-down view showing several crypts with biofilm. **b** Side-view showing biofilm inside crypt. **c** Shows a close up small bacterial aggregates outside the crypts located in the dashed box in image (**a**)

We examined colonic tissue from a patient with a right-sided colon cancer and as expected we found biofilm formation on the surface epithelium but surprisingly also deep into the crypts (Fig. 1). In addition, we also examined an adenoma tubular polyp from right colon and found no formation of bacterial biofilm (Fig. 2).

## Materials and methods

A conventional right-sided hemicolectomy was performed on a patient with an adenocarcinoma in the right colon. Tissue samples were obtained from resection margins. No preoperative oncological treatment or antimicrobial therapy. An adenoma tubular polyp from right colon was removed in routine colonoscopy. Samples were fixed in 4% buffered paraformaldehyde (pH 7.4), embedded in paraffin and then cut, deparaffinized and stained with PNA-FISH-Tamra5-conjugated universal bacterial (BacUni) 16S rRNA probe (AdvanDx, Woburn, MA, US). Slides were counter-stained with 3 µM 4′,6-diamidino-2-phenylindole (DAPI) (Life Technologies, Eugene, OR, US), mounted (Prolong Gold, Life technologies) and cover-slipped (Marienfield, Lauda-Königshofen, Germany). Slides were examined by CLSM (Axio Imager.Z2, LSM880 CLSM; Zeiss, Jena, Germany). Images was captured using 405 nm (DAPI) and 561 nm (Tamra-5) lasers, as well as a 488 nm for capturing the green auto fluorescence contour of the surrounding tissue as described elsewhere [7]. Images were further processed using IMARIS 9.2 (Bitplane, Zurich, Switzerland), using a complied “Easy 3D” mode of presentation.

## Discussion

To our knowledge, this is the first-time biofilm formation deep into colonic crypts are demonstrated in a patient with right-sided colon cancer. In theory, degradation of mucin by pathogens results in biofilm formation on the epithelial surface, invasion of potential driver bacteria into proliferating crypt compartments, adverse immune responses, accelerated proliferation and increased risk of deleterious mutations. Thus, biofilm invasion deep into colonic crypts would be expected to accelerate carcinogenesis. Also, bacteria like ETBF and *Fusobacterium Nucleatum* can impact the efficacy of conventional chemotherapy and checkpoint inhibitors. Biofilms in the right colon may explain some of the differences in treatment outcomes between right-sided and left-sided colon cancer. Current research on biofilm inhibitors and quorum sensing inhibitors may prove valuable in developing new drugs for future prevention and treatment of CRC.

## Abbreviations

APC: adenomatous polyposis coli
CPEC: colibactin-producing *Escherichia coli*
CRC: colorectal cancer
ETBF: enterotoxigenic *Bacteroides fragilis*
FAP: familial adenomatous polyposis
